# The effects of aerobic gymnastics training on performance-related variables in an elite athlete: a 2-year follow-up study

**DOI:** 10.3389/fphys.2024.1380024

**Published:** 2024-06-24

**Authors:** Oľga Kyselovičová, Erika Zemková

**Affiliations:** ^1^ Department of Gymnastics, Dance, Fitness & Combat Sports, Faculty of Physical Education and Sport, Comenius University in Bratislava, Bratislava, Slovakia; ^2^ Department of Biological and Medical Sciences, Faculty of Physical Education and Sport, Comenius University in Bratislava, Bratislava, Slovakia

**Keywords:** high-performance laboratory testing, balance and stability, flexibility, isokinetic leg muscle strength test, power, anaerobic test

## Abstract

This study investigates individual performance adaptations on 2 years of training between European Aerobics Championships. An elite, 22-year-old aerobic gymnast performed postural coordination test, Y-Balance test, squat and countermovement jumps, 60 s test of repeated jumps, an isokinetic leg muscle strength test, and the Wingate test. Postural stability and flexibility improved in terms of increased distance achieved in the Y-Balance test in the anterior (by 6.3%), posteromedial (by 2%), and posterolateral (by 4.8%) directions. Lower limb muscular endurance also increased, which can be corroborated by a reduced fatigue index in the 60 s test of repeated jumps (from 42% to 27% after the 1^st^ and to 22% after the 2^nd^ year of training). In addition, mean power increased during dominant (by 23.2% at 60°/s and by 18.5% at 180°/s) and non-dominant leg extension (by 4.9% at 180°/s and by 15.5% at 300°/s), plus dominant leg flexion (by 2.0% at 60°/s and by 6.9% at 300°/s). Similarly, peak torque/body weight ratio increased during dominant (by 24.9% at 60°/s, by 11.5% at 180°/s, and by 2.1% at 300°/s) and non-dominant leg extension (by 0.5% at 60°/s and by 6.4% at 300°/s), plus dominant leg flexion (by 1.7% at 60°/s and by 5.4% at 300°/s). However, 2 years of training failed to show any significant improvements in the explosive power of lower limbs and anaerobic performance. These findings indicate that general aerobic gymnastics training without any specific inputs leads to performance adaptation, namely, in abilities closely related to competition routine (dynamic balance and strength endurance of lower limbs).

## 1 Introduction

Gymnastics is a unique technical-aesthetic sport with a particular training process that demands technical perfection and a high level of physical conditioning (Sands et al., 2003). Qualities such as flexibility, speed, power, agility, strength, and balance are crucial for training (Bobo-Arce and Méndez Rial, 2013; Gateva, 2019) that direct gymnasts’ success. They relate to a gymnast’s ability to sustain injury-free and long-term participation in this sport (Sands et al., 2011). The development of motor skills through training includes the knowledge of specific skills determining the level of high performance, as well as methods assessing specific abilities (Gateva, 2016). Therefore, it is imperative to objectively measure and monitor an individual gymnast’s physical abilities.

So far, scientific research has mainly dealt with different physiological, functional, and technical aspects of Olympic disciplines, such as artistic, rhythmic and trampoline gymnastics (Poblano-Alcalá and Braun-Zawosnik, 2014; Rutkowska-Kucharska et al., 2018; Gateva, 2019; Urzela et al., 2020; Cabrejas et al., 2022; Líška and Kremnický, 2022; Vernetta, 2022; Farana et al., 2023; Jakše and Jakše, 2023). However, there are only a few studies published on non-Olympic discipline, aerobic gymnastics (Puiu and Dragomir, 2020).

Aerobic gymnasts perform short and intense routines either individually, in mixed pairs, in trios, or in groups without apparatus. The routines involve many complex movements, with different technical and artistic requirements. Gymnasts must demonstrate continuous movement patterns together with a variety of perfectly executed elements specified by technical guidelines in Code of Points (FIG, 2021). Different kinds of difficulty and acrobatic elements with precise take-offs, landings, and rotations performed in intense sequences are common demands in gymnastics (Abuwarda, 2020). Similarly, precise spatial and temporal coordination of multi-joint limb movements with postural control (Leskovec and Pavletič, 2019) is also required in aerobics. Considering the high-intensity movements and the short duration of the competitive routine (80 ± 5 s), the anaerobic metabolism is a determinant of aerobic gymnastics performance (Kyselovičová and Danielová, 2012; Aleksandreviciene et al., 2015).

The mastery of the technique of individual elements depends on the level of motor skills that ensure their implementation (Chernykh et al., 2021). Performance is very specific to each athlete. The improvement involves individualization based on a regular objective assessment of the adaptation, corresponding to the competition-specific effort characteristics, training loads, and changes in the process of motor development of each athlete (Lamošová et al., 2021). The gymnasts have to be prepared gradually over several years to sustain and develop their performance to succeed in competition (Stan et al., 2003; Fink and Hofman, 2015). Training demands and high-level competition performance emphasize the importance of controlling individual training. However, the variety and complexity of gymnasts’ events require not only different training approaches but also a wide range of physical and physiological assessments to monitor the progress of the gymnasts (Jemni, 2017). General and sport-specific testing represents an integral aspect of performance optimization in gymnastics (Mkaouer et al., 2017). This is ideal scenario, however, standardized tests close to sport-specific performance in gymnastics are very rare. A suitable alternative represents non-sport-specific field tests which also strongly correlate with the competition results in elite gymnasts (Vandorpe et al., 2012). Several attempts have been made to develop tests specific to aerobic gymnastics (Kyselovičová and Zemková, 2010a; Alves, 2015; Gateva, 2019). Despite the efforts of experts in recent years, a valid and reliable field test battery has not been developed yet. In addition, there is still not sufficient scientific data to provide robust optimal elite-level aerobic gymnast body composition and physiological profiles. This study aimed to investigate individual training adaptations on performance variables in an elite aerobic gymnast for 24 months, between the two European Championships.

## 2 Methods

### 2.1 Study design

The present study is a follow-up study to previous research by Kyselovičová et al. (2023). The main aim is to evaluate changes in physiological and performance variables of female elite aerobic gymnast. The entire observation period lasted 24 months (September 2021–September 2023). The athlete was instructed to train according to her training programs worked out by her coach before the research project and report their daily training for the whole 24-month period with no changes regarding the annual plan. She was tested on three occasions: beginning (T0), after 1^st^ year of training (T1), and after 2^nd^ year of training (T2). The T0 was set as a baseline, while T2 was the finish line. The study was designed based on multiple testing measurements. Measurements were conducted at the National Sports Centre Laboratory. Written informed consent and ethical committee approval of the Faculty of Physical Education and Sport, Comenius University in Bratislava, Slovakia (No. 1/2020) was obtained before the participation. The procedures followed were under the ethical standards on human experimentation stated in compliance with the 1964 Helsinki Declaration and its later amendments.

### 2.2 Subject

An elite female aerobic gymnast with the age of 22 years, height 166.5 cm, body mass 58.2 kg, and BMI 21.0 kg/m^2^ at baseline volunteered to participate in the study. She was a member of Slovakia’s national team and competed at the international level (World Cups, World and European Championships) in aerobic gymnastics for 3 years.

### 2.3 Testing procedures

To evaluate changes in performance variables, the gymnast was tested on three separate occasions as mentioned above (T0–T2). T0 was performed in September 2021, T1 in September 2022, and T2 in September 2023. All testing procedures were the same at all testing sessions. All tests were performed on 1 day. The athlete was instructed to do only light training the last 24 h before testing. The tests were also conducted at the same time of day in the morning to avoid circadian differences. The testing day started with anthropometric measurements. After analyzing the body composition by a multi-frequency bioelectrical impedance device InBody 770 (Cerritos, CA, United States), the gymnast performed a 15-minute self-conducted warm-up. Then she executed all tests in this order: the Imoove test; the Y-Balance test, followed by 5-min rest; the OptoJump Tests (squat jump–SJ, countermovement jump–CMJ, countermovement jump with arms–CMJarms), the 60-s jump test; the Biodex leg muscle strength test, and the Wingate test. There were additional rests after performing CMJarms (15 min), 60 s repeated jumps (15 min), and isokinetic leg strength test (20 min). Before each test, the gymnast was familiarized with the procedure. The main criteria for tests selection involved 1) assessment of overall abilities related to aerobic gymnastics skills and performance; 2) prediction and prevention of injuries (e.g.,: the Y-Balance test, the Imoove test); 3) higher validity and reliability compared to field tests. In addition, the tests were part of the Slovak Gymnastics Federation official test battery as an objective tool for national team members’ selection and evaluation.

#### 2.3.1 Postural coordination test

The postural coordination was assessed using the IMOOVE® system (Allcare Innovations, Chabeuil, France), a no-impact platform, based on an exclusive Ellisferic movement stimulating deep proprioception and vertebral movement to restore the muscular and postural balance of the body. The IMOOVE® system assesses the stability and coordination using the check-up program enabled by the presence of a monitor. The protocol (Di Corrado, 2023) is based on processing the results of information from pressure sensors placed in the stand plate, and from movable handles providing information about the reaction of the torso and upper limbs as a reaction to dynamic changes. IMOOVE® system was set to a dynamic mode, an intensity level of 3, a sensitivity level of 2, and a duration of evaluation of 60 s involving visual feedback in real time. The data used for the study included support and trunk stability, side distribution, and postural coordination. Assessment of individual parameters takes place on a scoring scale from 0 to 100 points, where a result of 100 is the best possible. All this information is presented as the final postural strategy index on a scale of 0–10.

#### 2.3.2 Lower quarter Y-Balance test

This test is a valid instrument to measure dynamic balance and postural control stability in anterior, posteromedial, and posterolateral directions. It has been performed according to the previously described protocol by Gribble et al. (2012). The test was conducted by the same procedure as presented by Kyselovičová et al. (2023). Briefly, the gymnast performed three trials, and the maximum reach in each direction was recorded. The results were calculated considering limb length to determine a “composite reach distance”.

#### 2.3.3 Isokinetic leg muscle strength test

The isokinetic leg muscle strength of knee extensors and knee flexors was measured using Biodex® System 3 (Biodex® Corporation, Shirley, New York, United States). Selecting low strength speed (60°/s), medium fast speed (180°/s), and high endurance speed (300°/s) as isokinetic testing speeds was essential for optimal strength assessment in gymnasts who utilize both strength and fast speeds (Baltzopoulos and Brodie, 1989). The test protocol consisted of a series of 5 repetitions at 60°/s, followed by 10 repetitions at 180°/s and 15 repetitions at 300°/s. The test was managed the same way as described in detail in previous study by Kyselovičová et al. (2023).

#### 2.3.4 Maximal vertical jump tests

Jumping performance was tested using optical time system (Optojump®, Microgate, Bolzano, Italy). The Optojump software (version 1.6) calculates the height of vertical jump from flight time using a simple method (9.81 × flight time^2^/8) described by Bosco (1983). The gymnast performed three separate jump tests in the following order: squat jump (SJ), countermovement jump (CMJ), and countermovement jump with arm swing (CMJarms). For the SJ tests, the knee-angle were 90°. No countermovements were allowed in this test, whereas no countermovement restrictions were given for the CMJ and CMJarms tests. The gymnast was given three consecutive attempts in each jump-test, and the best attempt was registered as the result.

#### 2.3.5 60 s test of repeated jumps

This test required the subject to execute maximal vertical rebound jumps while attempting to maintain the shortes possible ground contact time. Height of the jumps was measured using the same Optojump® (Microgate, Bolzano, Italy) as described above. Peak and mean power outputs for each jump were used to calculate a fatigue index (FI%) using the following formula: [(highest power output − lowest power output)/highest power output × 100] (Patterson et al., 2014). The blood lactate samples were taken in the 4^th^ minute following the 60 s test of repeated jumps.

#### 2.3.6 Wingate test

Frequent attempts of various jumps and other specific difficulty elements, including acrobatics, and highly intense aerobic movement patterns during the aerobic gymnastics routine indicate predominantly anaerobic energy contribution. For that reason, the standardized Wingate test which measures anaerobic performance has been used. After a standard warm-up, the subject performed a 30-s anaerobic Wingate test on a cycle ergometer (Isokinetic Cycle Ergometer–Monark 894E, Varberg, Sweden) to determine peak power, mean power, and fatigue index. The fatigue index was calculated by subtracting the minimal power from the maximal power, then divided by maximal power, and expressed as a percentage. The blood lactate samples were taken in the 5^th^ minute following the Wingate test. The test was performed according to the protocol described by Vandewalle et al. (1985).

## 3 Results

### 3.1 Changes in body composition over 2 years in an elite aerobic gymnast

The anthropometric characteristics of participants are summarized in Table 1. The athlete changed some of her body composition variables in terms of lightly increased BM (+1.6 kg), BMI (0.6 kg/m^2^), and BFM +1.4 kg. However, other anthropometrical and body composition parameters remained relatively unchanged over 2 years.

**TABLE 1:** Changes in anthropometric characteristics over 2 years in an elite aerobic gymnast.

Anthropometric variables	T0	T1	T2
Age (years)	22	23	24
Height (cm)	166.5	166.5	166.5
Body mass (kg)	58.2	59.8	59.8
Body mass index (kg/m^2^)	21	21.6	21.6
Skeletal muscle mass (kg)	26.9	27.4	26.9
Skeletal muscle mass (%)	46.2	45.8	45.0
Body fat mass (kg)	9.8	10.3	11.2
Body fat mass (%)	16.8	17.2	18.7
Right arm muscle mass (kg)	2.4	2.5	2.3
Left arm muscle mass (kg)	2.4	2.4	2.4
Trunk muscle mass (kg)	20.6	21	20.4
Right leg muscle mass (kg)	7.8	7.7	7.8
Left leg muscle mass (kg)	8.0	7.8	7.8
Body water (l)	35.5	36.2	35.6
Body water (%)	61	60.5	59.5
Extracellular water ratio	0.377	0.377	0.379
Proteins (kg)	9.6	9.8	9.6
Minerals (kg)	3.3	3.5	3.4
Basal metabolism (kcal)	1,416	1,439	1,420

T0–baseline, T1–after the 1^st^ year of training, T2–after the 2^nd^ year of training.

### 3.2 Changes in body balance and postural coordination during 2 years of training in an elite aerobic gymnast

The distance reached by both the D and ND leg in the Y-balance test increased by 6.3% in the anterior direction, by 2% in the posteromedial direction, and by 4.8% in the posterolateral direction (Table 2). The composite score of symmetry of both legs increased by 3.9%.

**TABLE 2:** Changes in body balance and postural coordination during 2 years of training in an elite aerobic gymnast.

Parameters of the Y-balance test	T0	T1	T2	% Changes T2 vs. T0
The distance reached by D leg in anterior direction (cm)	42	40	45.5	8.3
The distance reached by ND leg in anterior direction (cm)	58	53	60.5	4.3
The difference in distance reached by D and ND leg in anterior direction (cm)	16	13	15	−6.3
The distance reached by D leg in posteromedial direction (cm)	100	103	103	3.0
The distance reached by ND leg in posteromedial direction (cm)	106	108	107	0.9
The difference in distance reached by D and ND leg in posteromedial direction (cm)	6	5	4	−33.3
The distance reached by D leg in posterolateral direction (cm)	97	100	102	5.2
The distance reached by ND leg in posterolateral direction (cm)	102	107	106.5	4.4
The difference in distance reached by D and ND leg in posterolateral direction (cm)	5	7	4.5	−10
Composite score of the D leg symmetry (%)	98.35	100	103.09	4.8
Composite score of the ND leg symmetry (%)	109.47	110.29	112.76	3.0
Parameters of balance and postural coordination measured by IMOOVE system®				
Supports stability (pts)	100	100	88	−12
Supports distribution left and right (%)	50	50	49	−1
	50	50	51	+1
Trunk stability (pts)	62	77	78	+16
Trunk distribution left and right (%)	58	49	52	−6
	42	51	48	+6
Postural coordination (pts)	35	34	43	+8
Postural strategy index	7.6	8.2	8.1	+7.9

T0–baseline, T1–after the 1^st^ year of training, T2–after the 2^nd^ year of training, D–dominant, ND, non-dominant.

Regarding the IMOOVE® variables (Table 2), there was a 12% decrease in the support stability with a slight change in the left and right-side distribution (−1% and +1% respectively). The gymnast improved her trunk stability by 16% whilst the left and right distribution deteriorated (−6% and +6% respectively). In addition, postural coordination improved by 8%. Overall, postural strategy index increased by 7.9% (from an average value of 7.6 to a very good value of 8.1).

### 3.3 Changes in muscle strength and power during 2 years of training in an elite aerobic gymnast

The power and height of squat and countermovement jumps slightly decreased over 2 years (Table 3). However, fatigue index during a 60 s test of repeated jumps decreased from 42% to 27% after the 1st year of training and to 22% after the second year of training. This indicates a marked improvement of strength endurance of lower limbs over 2 years. Aerobic gymnast was able to maintain her maximal power while jumping much longer after 2 years of training compared to baseline level.

**TABLE 3:** Changes in muscle strength and power during 2 years of training in an elite aerobic gymnast.

Muscle strength and power parameters	T0	T1	T2	% Changes T2 vs. T0
Parameters of a squat jump				
Height of the jump (cm)	24.7	23.3	23.9	−3.2
Power (W/kg)	11.15	11.01	11.13	−0.2
Parameters of a countermovement jump				
Height of the jump (cm)	25.7	23.4	25.0	−2.7
Power (W/kg)	11.52	10.79	11.20	−2.8
Parameters of a countermovement jump with arm swing				
Height of the jump (cm)	30.2	29.2	29.2	−3.3
Power (W/kg)	12.47	12.80	12.62	1.2
Parameters of a 60 s test of repeated jumps				
Fatigue index (%)	42	27	22	−47.6
Post-exercise blood lactate (mmol/L)	3.68	5.05	4.35	18.2
Parameters of knee flexor/extensor strength measured by Biodex Isokinetic Dynamometer®				
Mean power during D and ND leg extension at 60°/s (W)	60.7	73.4	74.8	23.2
	82.9	80.1	78.0	−5.9
Mean power during D and ND leg flexion at 60°/s (W)	54.5	60.8	55.6	2.0
	60.0	80.1	59.5	−14.0
Mean power during D and ND leg extension at 180°/s (W)	120.3	120.8	142.5	18.5
	137.0	125.7	143.7	4.9
Mean power during D and ND leg flexion at 180°/s (W)	107.7	101.1	94.3	−12.4
	103.0	98.2	90.9	−11.7
Mean power during D and ND leg extension at 300°/s (W)	118.4	111.3	151.9	−6.0
	137.6	124.7	158.9	15.5
Mean power during D and ND leg flexion at 300°/s (W)	93.4	78.0	99.8	6.9
	94.8	84.5	94.5	−0.3
Total work during D and ND leg extension at 60°/s (J)	577.1	686.0	692.8	20.0
	777.6	764.6	698.1	−10.2
Total work during D and ND leg flexion at 60°/s (J)	501.4	563.1	512.8	2.3
	581.4	578.2	455.1	−21.7
Total work during D and ND leg extension at 180°/s (J)	861.7	879.5	961.6	11.6
	1,001.4	963.8	1,036.3	3.5
Total work during D and ND leg flexion at 180°/s (J)	768.0	765.6	674.1	−12.2
	815.6	782.5	686.2	−15.9
Total work during D and ND leg extension at 300°/s (J)	859.3	855.1	1,083.2	26.1
	1,019.5	990.0	1,223.5	20.0
Total work during D and ND leg flexion at 300°/s (J)	720.4	662.7	781.4	8.5
	800.0	747.9	782.5	−2.2
Peak torque/body weight ratio during D and ND leg extension at 60°/s	158.8	178.9	198.4	24.9
	208.5	205.9	209.5	0.5
Peak torque/body weight ratio during D and ND leg flexion at 60°/s	132.9	131.5	135.2	1.7
	143.5	133.2	117.3	−18.3
Peak torque/body weight ratio during D and ND leg extension at 180°/s	124.7	124.8	139.1	11.5
	147.7	143.9	147.1	−0.4
Peak torque/body weight ratio during D and ND leg flexion at 180°/s	104.0	99.3	91.1	−12.4
	104.8	102.8	89.7	−14.4
Peak torque/body weight ratio during D and ND leg extension at 300°/s	103.3	93.5	105.5	2.1
	108.2	107.4	115.1	6.4
Peak torque/body weight ratio during D and ND leg flexion at 300°/s	78.3	76.2	82.5	5.4
	85.6	79.0	83.2	−2.8

T0–baseline, T1–after the 1^st^ year of training, T2–after the 2^nd^ year of training, D–dominant, ND, non-dominant.

Regarding the isokinetic strength, mean power increased during leg extension at 60°/s by 23.2% and at 180°/s by 18.5% on the dominant leg whilst on the non-dominant at 180°/s by 4.9% and at 300°/s by 15.5% (Table 3). Mean power increased also during dominant leg flexion at 60°/s by 2.0% and at 300°/s by 6.9%. Similarly, peak torque/body weight ratio increased during leg extension at 60°/s by 24.9%, at 180°/s by 11.5% and at 300°/s by 2.1% on the dominant leg whilst on the non-dominant leg at 60°/s by 0.5% and at 300°/s by 6.4%. Peak torque/body weight ratio increased also during dominant leg flexion at 60°/s by 1.7% and at 300°/s by 5.4%.

### 3.4 Changes in anaerobic capabilities during 2 years of training in an elite aerobic gymnast

The anaerobic performance of an aerobic gymnast deteriorated, which may be corroborated by an increased fatigue index from 45.5% to 48.8% after the second year of training (Table 4).

**TABLE 4:** Changes in anaerobic capabilities during 2 years of training in an elite aerobic gymnast.

Wingate test parameters	T0	T1	T2	% Changes T2 vs. T0
Maximal power (W)	592.18	565.67	616.39	4.1
Maximal power (W/kg)	10.21	9.46	10.31	1.0
Mean power (W)	444.50	440.98	444.05	−0.1
Mean power (W/kg)	7.66	7.37	7.43	−3.0
Fatigue index (%)	42.51	44.41	48.82	14.8
Post-exercise blood lactate (mmol/L)	9.61	9.24	10.04	4.5

T0–baseline, T1–after the 1^st^ year of training, T2–after the 2^nd^ year of training.

## 4 Discussion

The present follow-up study examined exclusively the elite female aerobic gymnast, currently the most successful one representing Slovakia. Although the athlete was already considered at an elite level at the beginning of the study, she was not the best in the country at that time as successful as at the end of our observation. During the investigation period, the athlete had an average of 12–14 h of training per week. In addition, the athlete reported regular menstrual status and maintained the same overall dietary pattern throughout the whole 24 months.

Although our athlete was an adult gymnast, we emphasize that she maintained her body height and had slightly increased her body mass and BMI over the study period. The most marked anthropometric change was estimated in % BFM. The baseline value of 16.8%, which means a level “below” the norm, was shifted to the level of “normal” values of 18.7%. The relatively unchanged variables were segmental muscle mass of the limbs and trunk. These results are interesting in the sense that the athlete maintained, in addition to the overall dietary pattern, also the pattern of training. There is not yet a consensus regarding the desired ranges of anthropometric variables and body composition components outdated data for elite female aerobic gymnasts. It could be due to the different assessment technologies, the proper interpretation of the measured body composition, its connection with general nutritional intake, and the use of terms “elite-gymnast” or “high-performance gymnast” (Jakše and Jakše, 2023). However, it is known that success in high-level gymnastics compared with lower competitive levels is associated with smaller size, lower body mass, and body fat percentage (Bacciotti et al., 2017). An optimal amount of leg volume and leg mass contribute to success in elite gymnasts as well (Mohamed, 2011; Bastürg and Marangoz, 2018).

Postural control has been defined as the task of controlling one’s body position in space for the dual purposes of stability and orientation (Menant et al., 2021). Core stability is an important source of balance in sports activities (Kibler et al., 2006), and core muscle control is the basis for the successful technical performance of gymnastics balance exercises (Gateva, 2013). Compared with traditional training methods, core exercise is a new strength practice that could potentially improve skill performance among gymnasts (Luo et al., 2022). Indeed, the stronger core muscles not only more economically and harmoniously transfer the strength of athletes to the limbs, but also better maintain the stability of the trunk and hip joints so that gymnasts can show more coherent, coordinated, and stable complex technical movements (Yang and Li, 2010). In our study, we investigated trunk and lower limb stability with IMOOVE® system. The gymnast improved the ability to maintain an optimal posture on the balancing platform. However, despite increasing the postural strategy index of the gymnast over study period, it is necessary to focus on the further development of the perception of symmetry as the prolonged training time might induce structural changes in the gymnasts’ motion system (Douda et al., 2002) and affect asymmetries of lower limb due to the prevalence of exercises performed on the preferred side (Frutuoso et al., 2016). Improvement of posture and a feeling of symmetry can be beneficial in aerobic gymnastics as many of the difficulty elements require postural control and coordination (Kyselovičová and Zemková, 2010b). Such a suggestion is within Rodriguez et al. (2023) who demonstrated that core training should be included in training sessions to improve overall athletic performance. However, as underlined by several studies, training programs must respect the functional characteristics of the sports to be transferable (Myer et al., 2005; McGill, 2010). Thus, core strength exercises are functional for a specific sport when these exercises lead to an efficient and specialized motor unit recruitment to achieve the proper coordination of the segments involved in the kinetic chain of sport-specific skills (Lederman, 2010).

Gymnastics performance is based on symmetrical, both-sided movements (FIG, 2021). Moreover, it is crucial to understand what role asymmetries in both functional movement and isolated strength play in injury risk or when returning to sport after injury. Thus, in our study, dynamic balance using the Y-Balance test was assessed. While the differences between the D and ND legs at baseline were insufficient in two directions (anterior & posteromedial), a positive change was noticed at the end of the study with the overall result at a very good level. The differences in the ranges between the limbs were at the optimal level in posteromedial and posterolateral directions; however, the difference in the anterior direction reached significantly above the optimum level. Comparing the changes between T0 and T2, the gymnast achieved slightly higher percentage values of the total composite score for both limbs with the dominance of the left one at the end of the investigation. Persistent problems with the right ankle were probably a significant limitation in reaching better results in this test. On the positive side, at the end of the study, the gymnast achieved a composite score over 95% (103.09 for D leg and 112.76 for ND leg), which is associated with a low risk of injury (Schwiertz et al., 2020). Dynamic balance performance in athletes has a protective effect on lower extremity injuries (Butler et al., 2014; O’Malley et al., 2014). To improve dynamic balance, athletes should practice exercises that increase the isokinetic strength of knee circumference muscles (Aka and Altundag, 2020). This may be documented by the relationship between lower extremity muscle strength and balance performance (Muehlbauer et al., 2015; Myers et al., 2018). Therefore, it is obvious that lower extremity strength is important in displaying balance performance (Deniskina and Levik, 2001).

Regarding the isokinetic strength of our gymnast, the findings showed that a lateral deficit of D knee joint in extension at test speeds of 60°/s and 180°/s (strength and coordination) during the 2-year training has been eliminated at all. However, a strength deficit of D and ND knee joint extension and flexion at lower test speeds (60°/s and 180°/s) were still noticed at the end of the study. In addition, achieved values are below the limit of the recommended standard which is even more alarming. This can be because the competition routine focus is on turns, balances, and leaps rather than dynamic and static upper body skills (e.g., A-frames, helicopters, cuts, supports). Such elements are technically more difficult to perform, and the skill should be practiced more during training sessions. Additionally, turns and balances in a single-leg stance require a great amount of muscle concentric contraction at the hip, knee, and ankle to maintain body position. Thus, the time spent on the supporting leg is considerably greater. Furthermore, muscle imbalance of quadriceps/hamstring muscle groups at lower speeds was found in both D and ND legs. The finding follows Lanshammar and Ribom (2011), who demonstrated that in sports practice, a considerable asymmetry exists for the force relation between hamstrings and quadriceps in young adult females, with the hamstrings being weaker on the preferred limb and the quadriceps weaker on the non-preferred limb.

The importance of strength and power to successfully perform dynamic and varied gymnastics difficulty skills in sequences (Moeskops et al., 2019; Niespodzinski et al., 2021) has increased during the last decades. Core stability exercises performed in conjunction with plyometric exercises are also recommended to improve sports performance (Willardson, 2007). Accordingly, a variety of studies have been published using vertical jumps as a screening method and predictor of sports performance (Thomas et al., 2005).

The comparison of changes in squat and countermovement jumps variables showed that the gymnast’s power and height slightly decreased over 2 years. However, the fatigue index during a 60 s test of repeated jumps decreased, indicating a marked improvement in strength endurance of lower limbs, So, the aerobic gymnast was able to maintain her maximal power while jumping much longer after 2 years of training compared to baseline level. That improvement can be associated with the aerobic gymnastics routines’ character. Based on expertise and experience from gymnastics training, we can assume that the volume of rebound jumps in aerobic gymnastics training from a very young age may have an important, albeit different, influence on the dynamic and plyometric ability of gymnasts. Gymnasts need to reach a high level of their rate of force development in an extremely short time. This result could be explained by the fact that performance in aerobic gymnastics is realized mainly in standing, loading predominantly the lower part of the gymnasts’ body. In addition, the competitive routine must demonstrate continuous and complex intense movements, which require repeated dynamic rebounds (FIG, 2021) within approximately 80 min.

Considering the high-intensity movement and the total routine time, the anaerobic metabolism is one of the main determinants of aerobic gymnastics performance (Kyselovičová et al., 2012; Alves et al., 2015; Markov, 2020). Similarly, anaerobic metabolism comprises around 50% of energy contribution in rhythmic gymnastics (Guidetti et al., 2000). Greater anaerobic power is also required in artistic gymnastics with increased technical difficulty of acrobatic elements (Brooks, 2003; French et al., 2004; Jemni et al., 2006). Since plasma lactate levels following a simulated aerobic gymnastics competition are comparable to the lactate values after the lower-body Wingate test (Alves et al., 2015), the test is usually used for this purpose as shown by Jemni et al. (2006) in artistic and rhythmic gymnastics.

However, our investigation of gymnasts’ anaerobic performance showed no improvement, even deterioration over 2 years in the lower-body Wingate test. Therefore, it is questioned whether Wingate test is suitable to determine the specific anaerobic performance in aerobic gymnastics. This assumption may be corroborated by Wonisch et al. (2003) who demonstrated that it is almost impossible to reflect the specific muscular involvement and movement patterns of a particular sport in laboratory′s physical tests. Thus, low relationship between the real technical demands and the type of effort the gymnast performs in her specific competitive routines during the study period may be expected. Specific field tests in a form of repeated rebound jumps (Kyselovičová et al., 2010a) would be more appropriate to assess the specific aerobic gymnastics performance over the 80 s routine. From practical point of view, it would be interesting to know the relationships between test variables and competition results, representing mostly by execution and difficulty scores. However, this was not applicable in our study due to regular change of rules–Code of Point (between two Olympic cycles) over 2 years of investigation.

The study has some limitations related to the sample size; therefore, the results should be interpreted cautiously. Using laboratory tests instead of field tests to assess physiological variables to explain sport-specific performance changes is also questionable. Thus, the relationships between general abilities and specific performance in aerobic gymnastics should be part of future research.

The novelty of our study with three screenings of the successful elite aerobic gymnast under the same conditions not only enables interpretations of the results obtained but provides some practical recommendations:

1. Physical and physiological tests should be performed regularly as they provide useful information on the progress of gymnasts during long-term training.
2. To assess adaptation, it is necessary to assess individual performance changes during long-term training. The obtained results provide important feedback for the gymnast’s coach during the annual training plan.
3. Coaches should consider competition results (difficulty and execution scores) as adequate variables to correlate with the physical and physiological changes over time.
4. Regular basic screening for elite athletes is also recommended, as it provides data on muscle imbalance and thereby supports the decision-making process; for example, despite the improvement in the ability to maintain optimal body posture, it is necessary to focus on further development of the perception of symmetry.

The results of this study provide limited but initial support for a specific test battery to assess the performance in aerobic gymnastics. Although it may be a sign that some changes are needed, this kind of screening and monitoring has shown its usefulness because it is affordable and not time-consuming.

## 5 Conclusion

This follow-up study is one of the few that investigate a high-performance aerobic gymnast, competing internationally. Over the 24 months between the two European Aerobics Championships, an almost identical year-plan was used with 2-peak periodization while the athlete’s changes over the 2-year range were compared. Nevertheless, postural stability and flexibility in the anterior, posteromedial, and posterolateral directions improved. Jumping performance also markedly increased during a 60 s test of repeated jumps. In addition, isokinetic muscle strength during leg extension rather than leg flexion increased. However, there were no improvements in the explosive power of lower limbs and anaerobic capabilities over 2 years of training. From these findings, it is obvious that our elite aerobic gymnast improved in the ability to maintain balance in dynamic conditions and repeated rebounds which represent key abilities of aerobic competition routine.

## Data Availability

The raw data supporting the conclusions of this article will be made available by the authors, without undue reservation.
